# The Feasibility and Safety of Robot-Assisted Vaginal Natural Orifice Transluminal Endoscopic Surgery (RA-vNOTES) for Gynecologic Disease: 298-Case Series

**DOI:** 10.3390/healthcare13070720

**Published:** 2025-03-25

**Authors:** Qiannan Yang, Daniel Y. Lovell, Yingchun Ma, Chunhua Zhang, Xiaoming Guan

**Affiliations:** 1 Division of Minimally Invasive Gynecologic Surgery, Department of Obstetrics and Gynecology, Baylor College of Medicine, 6651 Main St., 10th Floor, Houston, TX 77030, USA; qiannan.yang@bcm.edu (Q.Y.); daniel.lovell@bcm.edu (D.Y.L.); 2 Department of Obstetrics and Gynaecology, The First Affiliated Hospital of Shandong First Medical University, No.16766 Jingshi Road, Jinan 250014, China; chinaiwa@163.com; 3 Department of Obstetrics and Gynaecology, The Second Affiliated Hospital of Nanjing Medical University, No. 121 Jiangjiayuan, Nanjing 210011, China

**Keywords:** robotic vNOTES, gynecologic disease, robotic surgery, feasibility

## Abstract

**Objectives:** To explore the feasibility and surgical outcomes of robot-assisted vaginal natural orifice transluminal endoscopic surgery (RA-vNOTES) for women suffering from gynecologic disease. **Methods:** We performed an observational study reporting and analyzing the perioperative outcomes of 298 patients with gynecologic disease who underwent RA-vNOTES in a single institution from June 2019 to August 2024. **Results:** A total of 298 patients with a median age of 41 years and median body mass index of 29 kg/m^2^ underwent RA-vNOTES. The primary indications for surgery were endometriosis (43.62%), chronic pelvic pain (11.07%), abnormal uterine bleeding (20.81%), and uterine leiomyomata (14.77%). A total of 286 of 298 (95.97%) patients had a hysterectomy. The median total operating time was 138 min, with a port placement time of 5 min, dock time of 3 min, and robot console time of 63 min. The median estimated blood loss was 50 milliliters. Endometriosis resection of all stages was performed in 192 of 298 (64.43%) patients. Three cases (1.01%) were converted to laparoscopic surgery. One case was converted to robot-assisted single incision plus one port laparoscopic surgery (SILS plus one) and two cases were converted to robot-assisted multi-port surgery. The total complication rate was 17.45% (52 cases), of which 2.1% (6 cases) were intraoperative complications and 15.44% (46 cases) were postoperative complications. **Conclusions:** Our findings indicate that RA-vNOTES is a feasible and less invasive option for various gynecologic procedures, including complex endometriosis excision and sacrocolpopexy.

## 1. Introduction

The pursuit of minimally invasive surgical techniques is a priority for patients and surgeons alike. Vaginal natural orifice transluminal endoscopic surgery (vNOTES) represents a significant advancement in minimally invasive surgery [1]. In contrast to traditional transabdominal laparoscopy, vNOTES employs the vaginal route for endoscopic procedures, enhancing cosmetic outcomes by eliminating abdominal incisions [2]. Combining single-port laparoscopic surgery (SPLS) with trans-vaginal surgery, vNOTES provides a safer, less invasive technique while improving upon the limitations of traditional transvaginal surgery [3]. vNOTES has proven to be safe and feasible in a number of gynecologic operations. Lee et al. conclude that performing a vNOTES hysterectomy is generally beneficial, citing short operative times and high patient satisfaction due to no abdominal incisions [4]. vNOTES has been employed for a vast array of gynecologic procedures including radical hysterectomy in cervical cancer, omentectomy in early-stage ovarian cancer, and even sentinel lymph node excision in patients with endometrial cancer [5,6,7,8,9]. Outside of gynecology, surgeons have performed appendectomies, cholecystectomies, bowel resections, splenectomies, and nephrectomies through the vagina [10,11,12,13,14]. However, traditional vNOTES has significant limitations. Restricted triangulation and spacing between surgical instruments provide challenges mainly in laparoscopic suturing, such as in sacrocolpopexy.

Another stride in minimally invasive surgery was the advent of the robotic platform. It was first approved for use in gynecologic surgery in 2005 by the Food and Drug Administration [15]. The robotic platform offers several benefits over traditional laparoscopy: enhanced three-dimensional visualization, articulating instrumentation, reduced surgeon tremors, and decreased surgeon fatigue [16]. With the use of wristed instruments, robot-assisted vNOTES (RA-vNOTES) improves the ability to proficiently suture and perform delicate pelvic dissection needed for complex surgery [17]. RA-vNOTES has been used to perform hysterectomy, myomectomy, sacrocolpopexy, and uterosacral ligament suspension surgeries [18,19,20]. We present 298 cases of varying procedures showcasing versatility, demonstrating the feasibility and surgical outcomes in utilizing RA-vNOTES.

## 2. Materials and Methods

### 2.1. Study Design

This study presents a retrospective case series involving 298 patients diagnosed with gynecologic pathology, observed from June 2019 to August 2024. These patients underwent RA-vNOTES for gynecologic indication at Baylor College of Medicine hospital affiliates (Baylor St. Luke’s Hospital and Texas Children’s Hospital). All surgical operations were performed by a fellowship-trained minimally invasive gynecologic surgeon (X. Guan). The Institutional Review Board at Baylor College of Medicine approved this study.

Medical data were identified by secure search through the surgical case log of the primary surgeon. The inclusion criteria consisted of patients who underwent RA-vNOTES using the Da Vinci Xi robotic system. There were no designated exclusion criteria. The data encompassed age, body mass index (BMI), race and ethnicity, medical history, surgical history, number of previous abdominal surgeries, and tobacco consumption. The primary outcomes of interest were intraoperative and postoperative complications. The Clavien–Dindo classification was used for postoperative complications, which included grade I to grade V. Grade I—any deviation from the normal postoperative course without the need for pharmacological treatment, or surgical, endoscopic, and radiological interventions. Allowed therapeutic regimens are as follows: drugs as antiemetics, antipyretics, analgesics, diuretics and electrolytes, and physiotherapy. This grade also includes wound infections opened at the bedside. Grade II—requiring pharmacological treatment with drugs other than listed for grade I complications, blood transfusions, and total parenteral nutrition. Grade III—requiring surgical, endoscopic, or radiological intervention. Grade IV—life-threatening complications (including central nervous system complications) requiring management from the intensive care unit (ICU). Grade V—death of a patient [21]. Secondary surgical outcomes included total operative time (colpotomy initiation to vaginal cuff closure), port placement time, robot dock time, robot console time, estimated blood loss, conversion to laparotomy or abdominal laparoscopy, length of hospitalization (from the time of surgery to the time of discharge). Additional information was collected including the indication for surgery, and number and type of procedures performed. Patients were routinely evaluated in the clinic 3–6 weeks postoperatively. At this time, each patient completed a pain questionnaire rating their postoperative pain levels at week one, two, and three.

### 2.2. Surgical Technique

After the administration of general endotracheal anesthesia, the patient was positioned in dorsal lithotomy position with the arms secured alongside the body. Two grams of cefazolin is typically administered as the pre-operative antibiotic. If bowel surgery is planned, one gram of ertapenem is given in lieu of cefazolin. At times, unforeseen bowel endometriosis is visualized once intra-peritoneal. In these cases, one gram of ertapenem is given in addition to the cefazolin. It is typically our practice to place bilateral temporary ureteral stents with indocyanine green (ICG) injection for patients who undergo RA-vNOTES at the beginning of the procedure. Following stent placement, a Foley catheter is placed for the duration of surgery.

#### 2.2.1. RA-vNOTES Hysterectomy

**Figure 1:**
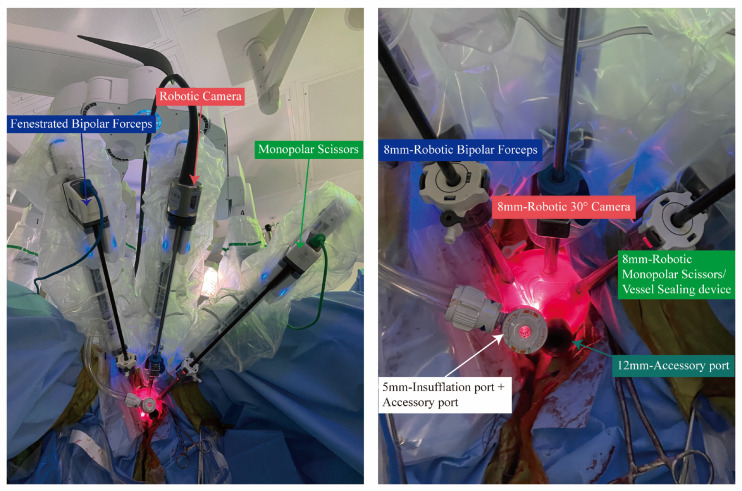
Da Vinci Xi vNOTES port placement.

The standard steps for a vaginal hysterectomy are followed when performing an RA-vNOTES hysterectomy. Due to adhesions, a complete hysterectomy may not be possible, but ideally both uterine artery pedicles would be secured. Once it is no longer feasible to continue the hysterectomy transvaginally, a vaginal port is placed. A 0-polyglactin suture is tied between the posterior and anterior colpotomy at the 10, 2, 4, and 8 o’clock positions, and sutured to the GelPOINT Mini^®^ advanced access port (Applied Medical, Rancho Santa Margarita, CA, USA) ring. In the GelPOINT, a 5-mm AirSeal^®^ System trocar (CONMED, Utica, NY, USA), one 12-mm GelPOINT advanced access trocar, and three 8-mm robotic trocars are placed. The Da Vinci Xi robotic system (Intuitive Surgical, Sunnyvale, CA, USA) is then docked (Figure 1) on the side of the patient, with arms oriented in an “upper abdominal view”. The remaining steps of the hysterectomy are completed with the use of robotic assistance. Additional indicated procedures such as excision of endometriosis, oophorectomy, oophoropexy, ovarian cystectomy, or high uterosacral ligament suspension are then performed. Following completion of the procedure, the robot is undocked. The specimens are removed transvaginally, the vaginal port is removed, and the vaginal cuff is closed transvaginally in a continuous fashion using a barbed suture.

#### 2.2.2. RA-vNOTES Surgeries Without Hysterectomy

In RA-vNOTES without a hysterectomy, a two-centimeter posterior colpotomy is created with the apices sutured to prevent an extension. The GelPOINT Mini device is then placed along the vagina and through the colpotomy with the same trocar arrangement as seen in a hysterectomy procedure. The abdomen is insufflated followed by docking the Da Vinci Xi robot. Procedures such as myomectomy, high uterosacral ligament suspension, ovarian cystectomy, or salpingectomy can then be completed with the robotic platform. Similarly, once the procedure is complete, the robot is undocked, the specimens are removed transvaginally, and the posterior colpotomy incision is closed with incorporation of the parietal peritoneum using a 0-barbed suture.

### 2.3. Data Analysis

All continuous variables were tested for normality using descriptive statistics for skewness and kurtosis, visual evaluation of histograms, and the Kolmogorov–Smirnov test. As our primary outcome did not have a normal distribution, all continuous data are described as medians [inter-quartile range (IQR)]. Groups were compared by Mann–Whitney U-test for non-normal distribution. Categorical data are reported as proportions and percentages, with analysis using Fisher’s exact test. All statistical analyses were carried out using SPSS software (version 25.0; SPSS Inc., Chicago, IL, USA), and the level of significance was set at *p* < 0.05.

## 3. Results

A total of 298 patients underwent RA-vNOTES from June 2019 to August 2024. Table 1 provides a detailed overview of the characteristics of these patients. The median age was 41 [IQR 36–46] years. The median BMI was 29 [IQR 24–35] kg/m^2^. Self-reported race and ethnicity was as follows: 53.69% Caucasian, 20.81% African American, 14.09% Hispanic, and 11.41% Asian. The percentage of tobacco users was 11.07%. The median gravidity and vaginal deliveries were 2 [IQR 1–3] and 0 [IQR 0–2], respectively. A total of 12% of patients had prior cervical surgeries and 76% of patients had previous abdominal surgeries.

**Figure 2:**
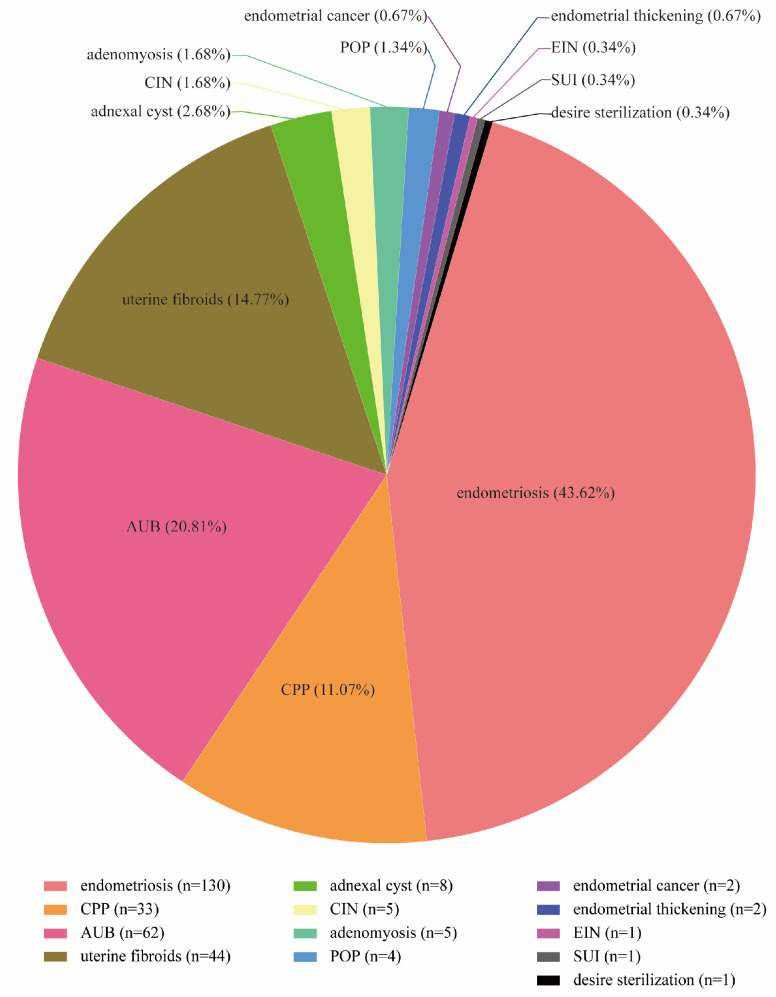
Distribution of primary surgery indications. CPP: chronic pelvic pain; AUB: abnormal uterine bleeding; CIN: cervical intraepithelial neoplasia; POP: pelvic organ prolapse; EIN: endometrial intraepithelial neoplasia; SUI: stress urinary incontinence.

The primary indications for surgery not only included endometriosis (43.62%), chronic pelvic pain (11.07%), abnormal uterine bleeding (20.81%), uterine fibroid (14.77%), and other benign gynecologic pathology, but also included cervical intraepithelial neoplasia (1.68%), endometrial intraepithelial neoplasia (0.34%), and endometrial cancer (0.67%), which are shown in Figure 2. The classification of chronic pelvic pain does not exclude endometriosis; it encompasses patients for whom prior advanced imaging (MRI) did not indicate endometriosis or for whom no previous surgical report documented endometriosis. Chronic pelvic pain is diagnosed when no other objective data support a more specific diagnosis, and symptoms have persisted for more than six months. The indication for RA-vNOTES in 33 of 298 (11.07%) patients was chronic pelvic pain. The presence of endometriosis was confirmed by pathology in 26 out of 33 patients, representing 78.79% of the cohort.

**Figure 3:**
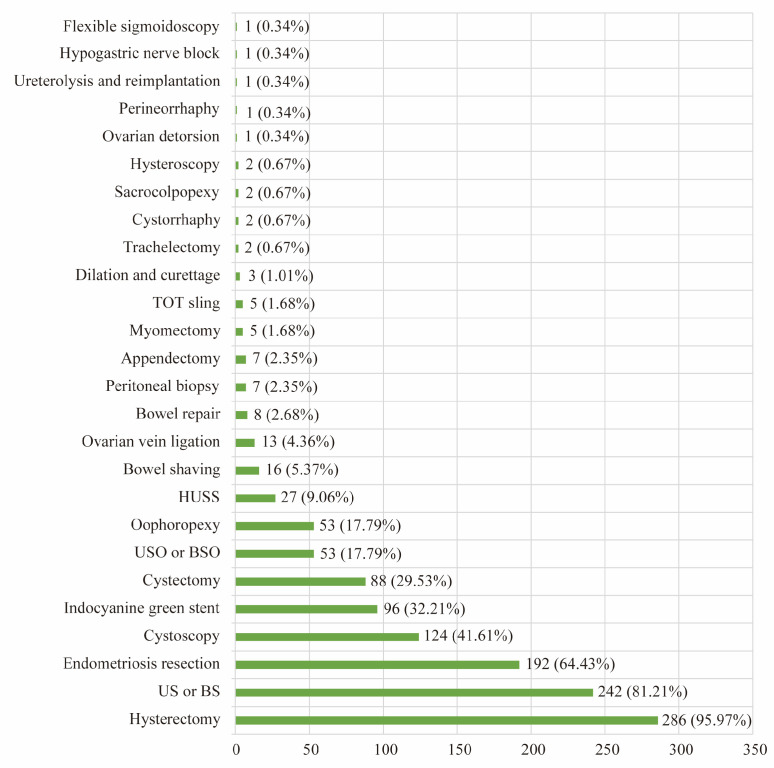
The performed surgical procedures. US or BS: unilateral or bilateral salpingectomy; USO or BSO: unilateral or bilateral salpingo-oophorectomy; HUSS: high uterosacral ligament suspension; TOT sling: transobturator tape sling.

Table 2 provides an overview of surgical outcomes. The median number of procedures performed was four [IQR 3–6], (detailed in Figure 3) with 286 of 298 (95.97%) patients undergoing hysterectomy. The median total operative time was 138 [IQR 116–167] minutes, with port placement time of 5 [IQR 3–7] minutes, robot dock time of 3 [IQR 2–5] minutes, and robot console time of 63 [IQR 40–87] minutes. The median estimated blood loss was 50 [IQR 25–50] milliliter. Endometriosis resection was performed in 192 of 298 (64.43%) patients. According to the American Society for Reproductive Medicine (ASRM) endometriosis classification, 34.56% of patients had stage I endometriosis, 12.75% with stage II, 7.38% with stage III, and 9.73% with stage IV. A total of 69.13% of patients were discharged within 24 h. Conversion to an abdominal procedure was seen in 1.01% of the patients. One case was converted to robot-assisted single incision plus-one port laparoscopic surgery (SILS plus one) and two cases were converted to robot-assisted multi-port surgery.

Table 3 presents the pain scores recorded from 254 patients prior to, and following surgery. Forty-four patients were excluded due to not completing the pain questionnaire at their follow-up visit. Patients with a history of preoperative pain exhibited statistically significant higher pain scores at week 1, 2, and 3 post-surgery, compared to those without preoperative pain (*p* < 0.001). Furthermore, patients with statistically significant preoperative pain had significantly reduced pain scales at week 1, 2, and 3 following surgery compared to their self-reported preoperative scores (*p* < 0.001), as illustrated in Table 4.

The total complication rate was 16.78% (50 cases), of which 1.34% (4 cases) were intraoperative complications, and 15.44% (46 cases) were postoperative complications, as detailed in Table 5. We classified postoperative complications by the Clavien–Dindo (CD) grade. Five patients (1.68%) had grade I, 35 patients (11.74%) with grade II, and 6 patients (2.01%) with grade III. We observed no CD-grade IV and V complications. Overall, urinary tract infection was the most common postoperative complication, with a rate of 9.73%. When patients were subgrouped based on whether they underwent a hysterectomy or not, the complication rates showed no significant difference between the two groups.

## 4. Discussion

Our study demonstrates that RA-vNOTES with the Da Vinci Xi platform is safe and effective in several gynecologic surgeries, particularly complex procedures such as endometriosis surgery. To our knowledge, this is the largest case series that demonstrates the use of RA-vNOTES using the Da Vinci Xi platform, in several gynecologic diseases. In recent years, with the advancement of vNOTES technology, an increasing number of gynecologists have begun using robotic systems to perform transvaginal natural orifice surgery. In addition to our team’s reports, numerous studies on the use of RA-vNOTES in gynecology have been published by authors from various countries, including those from developing nations [22,23,24]. Based on our team’s experience, we recommend that surgeons with less experience begin with simple RA-vNOTES hysterectomy before progressing to more complex cases after completing the learning curve. Previous reports suggest that the RA-vNOTES learning curve requires approximately 10 cases of RA-vNOTES hysterectomy and 10–20 cases in port placement and robotic docking to achieve proficiency [25].

Several studies have shown the diverse procedures and indications for vNOTES. Previous investigations on vNOTES indicate that most of these procedures were limited to hysterectomy and adnexal surgery, which are generally performed using traditional approaches. A prior study of 1000 vNOTES performed by a single surgeon presented complication rates related to certain procedures: 73% hysterectomies, 18% adnexal surgeries, 4% salpingectomies, 3% ovarian cystectomies, 1% myomectomies, and 1% other procedures (including appendectomy, omentectomy, and adhesiolysis) [26]. Hurni reported a study on the early surgical outcomes of vNOTES for benign gynecologic indications. In that study of 550 patients, 66.4% underwent a vNOTES hysterectomy, 30.4% had a procedure limited to the adnexa, and 3.3% received other interventions, including myomectomy, pelvic adhesiolysis, post-hysterectomy pelvic hematoma drainage, pelvic organ prolapse repair, and appendectomy [27]. However, robotics offers the ability to perform more complex surgeries, such as endometriosis resection and sacrocolpopexy. In our study, the primary diagnoses not only included benign diseases such as endometriosis (43.62%), chronic pelvic pain (11.07%), abnormal uterine bleeding (20.81%), uterine fibroid (14.77%), and pelvic organ prolapse (1.34%), but also included cervical intraepithelial neoplasia (1.68%), endometrial intraepithelial neoplasia (0.34%), and endometrial cancer (0.67%). Of the 298 patients, 286 (95.97%) had a hysterectomy performed, of which 192 (64.43%) patients underwent endometriosis resection. Of those undergoing endometriosis resection 34.56% of patients had stage I endometriosis, 12.75% with stage II, 7.38% with stage III, and 9.73% with stage IV. In the future, advancements in robotic technology may further expand the applications of vNOTES, allowing for an even broader range of minimally invasive procedures.

The median number of procedures performed for each case was four. The median operative time was 138 min, which is in the range of 108–157 min as previously reported for RA-vNOTES hysterectomy [23,24,28]. The estimated blood loss in the case series was minimal and was consistent with previous studies on robotic and vNOTES approaches [29,30,31]. The median length of hospitalization, calculated from the time of surgery to the time of discharge, was 0 days (IQR 0–1 days). A total of 69.13% of patients were discharged within 24 h, which is shorter compared with previous studies [24,32]. In our case series, the pain scales of patients with significant preoperative pelvic pain were statistically significantly higher in the following three weeks compared to those without preoperative pain. Thus, clinicians should be aware of patients who have a history of endometriosis, or preoperative pain, and set expectations for the recovery process during preoperative counseling.

A recent study comparing robot-assisted versus traditional vNOTES hysterectomy found that in 35 RA-vNOTES cases, there were no conversions. However, 6 of 79 (7.6%) cases performed via traditional vNOTES were converted to a transabdominal approach because of poor visualization, inability to achieve hemostasis, presence of a large broad ligament fibroid or noticeably enlarged uterus, or posterior cul-de-sac obliteration [27]. The previously reported conversion rate of RA-vNOTES varied from 0% to 2.38% [3,24,25]. In our study, three cases (1.01%) were converted to laparoscopic surgery. The first case to be converted to robot-assisted SILS plus one was due to unforeseen obliteration of the posterior cul-de-sac. This required an unplanned bowel resection by colorectal surgery with primary anastomosis due to deep infiltrating endometriosis of the rectum. The second case to convert was to a robot-assisted multi-port surgery because of posterior peritoneal entry complicated by rectal injury. This case required unplanned intra-operative consult to colorectal surgery to perform a colporrhaphy. The third case to convert was to a robot-assisted multi-port surgery due to resection of stage IV endometriosis complicated by transection of the right ureter. Urology was consulted to perform ureterolysis, and ureteroureterostomy. In response to this incident, we implemented a standardized procedure for vaginal surgery. At the onset of the procedure, cystoscopy is performed and temporary bilateral ureteral stents are placed with injection of ICG. Since the implementation, we have not encountered any further cases of ureteral injury in over 100 patients with endometriosis, including more than 20 cases with stage IV endometriosis.

Though colpotomy differs between patients with and without a hysterectomy, no significant difference in complication rates was found between the two groups. When considering intraoperative complications associated with our study, 2 of 298 (0.67%) patients had bladder injury, 1 of 298 (0.34%) patients had a ureteral injury, and 1 of 298 (0.34%) patients had a bowel injury. Hurni observed three cases (0.5%) of bowel injury associated with both vNOTES hysterectomies and adnexal procedures [26]. Two studies reported a bladder injury rate of 1.6% and 1.2% over 550 and 730 vNOTES hysterectomies for benign indications, respectively [26,27]. A study involving 839 women who underwent transabdominal hysterectomy, vaginal hysterectomy, or laparoscopic-assisted vaginal hysterectomy revealed a bladder injury rate of 2.9% (24 out of 839 cases) and a ureteral injury rate of 1.8% (15 out of 839 cases), with transection and kinking identified as the most common types of injury [33]. Additionally, one review article on 1337 patients who underwent laparoscopic treatment for ureteral endometriosis found that the prevalence of ureteral injury was 3.1% [34]. There are two reasons why the ureteral injury was much lower in our study. First, RA-vNOTES might help reduce ureteral injuries due to better visualization when approximating distance between the ureters and the vaginal apex. Second, we implemented a protocol for ICG stent placement making the ureters easily identifiable.

In regards to postoperative complications in our study, urinary tract infection emerged as the most significant occurring at a rate of 9.73% (29 out of 298 cases), consistent with the rate reported in a prior study. Their data demonstrated a rate between 8.33% and 12.4% [25,35]. As for postoperative surgical site infections, 5 of 298 (1.68%) patients had a superficial surgical site infection, all diagnosed to be vaginal cuff cellulitis. Only 1 of 298 (0.34%) patients had a deep surgical site infection, diagnosed to be a pelvic infection. Additionally, 6 of 298 (2.01%) patients had a reoperation: one patient with a pelvic abscess—requiring a transvaginal pelvic abscess drain by interventional radiology; one patient had a diagnostic laparoscopy—for a small bowel volvulus; one patient with persistent vaginal cuff granulation tissue—requiring excision and repair of the vaginal cuff; one patient had a vaginal cuff repair because of vaginal cuff bleeding; and two patients had a vaginal cuff revision secondary to a vaginal cuff hematoma following evacuation.

The cost-effectiveness of RA-vNOTES is a crucial consideration in the decision-making process for hospitals and surgeons. Compared to laparoscopic surgery, the total expenses of robot-assisted surgery were $2189 to $6685 more per case [36,37]. Despite the significantly higher burden of comorbidities in patients undergoing robotic surgery compared to laparoscopy, robotic surgery offers several advantages. It is particularly beneficial for patients with conditions such as diabetes, hypertension, cardiovascular disease, renal disease, obesity, morbid obesity, and pulmonary disease. Additionally, robotic surgery is associated with shorter hospital stays [37].

When compared to robot-assisted multiport surgery, the total expenses of robot-assisted single-site surgery were $1864 less per case [38]. RA-vNOTES is a type of robot-assisted single-site surgery that may cost less than robot-assisted multi-port surgery. Additionally, the robot enabled our group to perform more complex surgeries in our study, such as endometriosis excision in 65% of patients, a procedure that is typically thought of as difficult and challenging with traditional laparoscopic surgery. With the aforementioned, if a benign gynecologist is considering robot-assisted laparoscopic surgery for a patient, RA-vNOTES should also be considered as a viable option for achieving “incisionless surgery”. There is a need for further research to compare the cost of RA-vNOTES with traditional vNOTES or robot-assisted single-site surgery.

The strengths of this study are the generalizability of the protocol and robotic platform for a myriad of procedures. Through the experience of one surgeon, progressively more challenging and complex procedures were planned. Additionally, the large sample size in our series allows for patterns to develop in complication rates, protocolization, and confidence in procedures performed. However, the limitations of this study must be acknowledged. First, the retrospective nature of the study introduces potential biases in data collection and analysis. Second, its single-center cohort observational design lacked a control group. Third, all procedures were performed by a single highly experienced surgeon, which may limit the generalizability of the findings. Additional research is necessary in a multi-center context with an expanded sample size to confirm and generalize these findings.

## 5. Conclusions

Our data suggests that RA-vNOTES is a feasible surgical approach in expert hands for a variety of gynecologic diseases. As technology continues to evolve, RA-vNOTES is likely to become an increasingly integral part of gynecologic surgery, with the potential to improve patient care and expand the possibilities of minimally invasive procedures.

## Figures and Tables

**Table 1:** Patients’ characteristics (N = 298).

Characteristic	Median [IQR] or N (%)
Age, years	41 [36–46]
Body mass index, kg/m^2^	29 [24–35]
Race and ethnicity	
Caucasian	160 (53.69)
African American	62 (20.81)
Asian	34 (11.41)
Hispanic	42 (14.09)
Tobacco use	33 (11.07)
Gravidity	2 [1–3]
Vaginal deliveries	0 [0–2]
History of cervical procedure	
0	262 (87.92)
1	35 (11.74)
2	1 (0.34)
Number of previous abdominal surgeries	
0	71 (23.83)
1	76 (25.50)
2	70 (23.49)
3 or more	81 (27.18)

Notes: IQR: inter-quartile range.

**Table 2:** Patients’ surgical outcomes (N = 298).

Variables	Median [IQR] or N (%)
Number of procedures performed	4 [3–6]
Total operative time, min	138 [116–167]
Port placement time, min	5 [3–7]
Robot dock time, min	3 [2–5]
Robot console time, min	63 [40–87]
Estimated blood loss, mL	50 [25–50]
Additional endometriosis resection	
Yes	192 (64.43)
Stage I	103 (34.56)
Stage II	38 (12.75)
Stage III	22 (7.38)
Stage IV	29 (9.73)
No	106 (35.57)
Length of hospitalization, days	0 [0, 1]
0	206 (69.13)
1	81 (27.18)
2	8 (2.68)
3	2 (0.67)
4	1 (0.34)
Conversion	3 (1.01)

Notes: IQR: inter-quartile range.

**Table 3:** Preoperative and postoperative pain scales (N = 254).

Postoperative Pain,	History of Preoperative Pain		*p* Value
Median [IQR]	No (88)	Yes (166)	
Week 1	4 [2–6]	6 [4–8]	<0.001
Week 2	2 [2–4]	4 [2–6]	<0.001
Week 3	1 [0–2]	2 [1–4]	<0.001

Notes: IQR: inter-quartile range.

**Table 4:** Preoperative and postoperative pain scales (N = 166).

	Median [IQR]	*p* Value
Preoperative pain scores	9 [8–10]	
Postoperative pain scores		
Week 1	6 [4–8]	<0.001
Week 2	4 [2–6]	<0.001
Week 3	2 [1–4]	<0.001

Notes: IQR: inter-quartile range.

**Table 5:** Surgical complications (N = 298).

Complications	Total (N = 298)	Hysterectomy (N = 286)	No Hysterectomy (N = 12)	*p* Value
**Total**	**50 (16.78)**	**48 (16.78)**	**2 (16.67)**	**1.00**
Intraoperative complications	**4 (1.34)**	**3 (1.05)**	**1 (8.33)**	**0.15**
Bladder injury	2 (0.67)	2 (0.70)	0	
Ureter injury	1 (0.34)	1 (0.35)	0	
Bowel injury	1 (0.34)	0	1 (8.33)	
**Postoperative complications**	**46 (15.44)**	**45 (15.73)**	**1 (8.33)**	**0.70**
*CD grade I*	*5 (1.68)*	*5 (1.75)*	*0*	
Difficulty ambulating	1 (0.34)	1 (0.35)	0	
Fever and cough	3 (1.01)	3 (1.05)	0	
Nausea and dizziness	1 (0.34)	1 (0.35)	0	
*CD grade II*	*35 (11.74)*	*34 (11.89)*	*1 (8.33)*	
Urinary tract infection	29 (9.73)	28 (9.79)	1 (8.33)	
Vaginal cuff cellulitis	5 (1.68)	5 (1.75)	0	
Pelvic infection	1 (0.34)	1 (0.35)	0	
*CD grade III (Reoperation)*	*6 (2.01)*	*6 (2.10)*	*0*	
Vaginal cuff hematoma	2 (0.67)	2 (0.70)	0	
Pelvic abscess	1 (0.34)	1 (0.35)	0	
Small bowel volvulus	1 (0.34)	1 (0.35)	0	
Vaginal cuff granulation tissue	1 (0.34)	1 (0.35)	0	
Vaginal cuff bleeding	1 (0.34)	1 (0.35)	0	

Notes: CD grade: Clavien–Dindo classification grade. Values are given as N (%) (Fisher exact test).

## Data Availability

The data that support the findings of this study are available on reasonable request to the corresponding author. The data are not publicly available due to privacy or ethical restrictions.

## Abbreviations

The following abbreviations are used in this manuscript:

RA-vNOTES	Robot-Assisted Vaginal Natural Orifice Transluminal Endoscopic Surgery
BMI	body mass index
SILS	single incision laparoscopic surgery
vNOTES	vaginal natural orifice transluminal endoscopic surgery
ICG	indocyanine green
IQR	inter-quartile range
CD-grade	Clavien–Dindo grade
